# Proteomics computational analyses suggest that the carboxyl terminal glycoproteins of Bunyaviruses are class II viral fusion protein (beta-penetrenes)

**DOI:** 10.1186/1742-4682-1-10

**Published:** 2004-11-15

**Authors:** Courtney E Garry, Robert F Garry

**Affiliations:** 1 Department of Microbiology and Immunology, Tulane University Heath Sciences Center, 1430 Tulane Avenue, New Orleans, Louisiana 70112 USA

**Keywords:** viral fusion proteins, Bunyavirus envelope glycoproteins, proteomics computational analyses, glycoprotein structure, virus evolution

## Abstract

The Bunyaviridae family of enveloped RNA viruses includes five genuses, orthobunyaviruses, hantaviruses, phleboviruses, nairoviruses and tospoviruses. It has not been determined which Bunyavirus protein mediates virion:cell membrane fusion. Class II viral fusion proteins (beta-penetrenes), encoded by members of the Alphaviridae and Flaviviridae, are comprised of three antiparallel beta sheet domains with an internal fusion peptide located at the end of domain II. Proteomics computational analyses indicate that the carboxyl terminal glycoprotein (Gc) encoded by Sandfly fever virus (SAN), a phlebovirus, has a significant amino acid sequence similarity with envelope protein 1 (E1), the class II fusion protein of Sindbis virus (SIN), an Alphavirus. Similar sequences and common structural/functional motifs, including domains with a high propensity to interface with bilayer membranes, are located collinearly in SAN Gc and SIN E1. Gc encoded by members of each Bunyavirus genus share several sequence and structural motifs. These results suggest that Gc of Bunyaviridae, and similar proteins of Tenuiviruses and a group of *Caenorhabditis elegans *retroviruses, are class II viral fusion proteins. Comparisons of divergent viral fusion proteins can reveal features essential for virion:cell fusion, and suggest drug and vaccine strategies.

## Introduction

Two classes of viral envelope proteins that mediate virion:cell fusion have been described. Class I and II fusion proteins (aka α-and β-penetrenes) are distinguished, in part, by the location of the "fusion peptide," a cluster of hydrophobic and aromatic amino acids that appears critical for fusing viral and cell membranes. The fusion peptides of class I fusion proteins are located at or near the amino terminus, whereas fusion peptides of class II fusion proteins are internal. The overall structures of these two classes of viral fusions proteins are also distinct. Class I fusion proteins have a pair of extended α helices that are separated by sequences variable in length, but usually containing one or more dicysteine linkages. Several otherwise disparate viruses, including orthomyxoviruses, paramyxoviruses, retroviruses, arenaviruses, filoviruses and coronaviruses encode class I fusion proteins [1-4]. Class II fusion proteins are comprised mostly of antiparallel β sheets. The prototypic class II fusion protein is the E glycoprotein of tick-borne encephalitis virus (TBEV), a member of the genus flavivirus of the Flaviviridae family [5]. E possesses three β sheet domains (I-III). In the slightly curved rod-like configuration of the E protein present in the virion, the fusion peptide is located at the tip of domain II, the furthest point distal from the C-terminal transmembrane anchor. The virion configuration of envelope glycoprotein E1, the fusion protein of the Alphavirus Semliki Forest virus (SFV), demonstrates a remarkable fit to the scaffold of TBEV E [6]. E of dengue virus (DEN) and West Nile virus, medically important flaviviruses, also can be fit to the class II structure [7,8]. Recent studies indicate that TBEV E, DEN E and SFV E1 undergo similar conformational changes upon exposure to low pH, as encountered during entry via endocytic vesicles, suggesting a common fusion mechanism [9-11]. Based on sequence similarities, it is likely that E1 of other Alphaviruses and E of other members of the flavivirus genus within the family Flaviviridae are also class II fusion proteins. Members of the two other genuses in the Flaviviridae, hepaciviruises and pestiviruses, appear on the basis of proteomics computational analyses to encode truncated class II fusion proteins [12].

The Bunyaviridae family of enveloped RNA viruses includes five disparate genuses. Orthobunyaviruses, phleboviruses, nairoviruses and tospoviruses are spread by insect vectors, whereas hantaviruses are spread by rodent vectors [13]. Members of each Bunyavirus genus include important human and animal pathogens, except the tospoviruses, whose members infect plants [14,15]. The Bunyavirus genome consists of three single-stranded RNA segments. The envelope glycoproteins are encoded by the middle-sized segment (M) [16,17]. Members of each genus encode two glycoproteins that are present on the virion surface, and designated Gn and Gc to refer to their location amino terminal or carboxyl terminal on the M encoded polyprotein. The M segments of orthobunyaviruses, phleboviruses, and tospoviruses have been shown to encode for "nonstructural" proteins (NSm). In the case of the orthobunyaviruses and phleboviruses, NSm is synthesized as part of the polyprotein, but in tospoviruses NSm is encoded via an ambisense strategy by a separate mRNA [18]. The identity and structure of Bunyavirus fusion protein(s) are unknown, though it is likely that Gn or Gc fulfills this role. Proteomics computational analyses suggest that Bunyavirus Gc, and similar proteins of Tenuiviruses and a group of *Caenorhabditis elegans *retroviruses, are class II viral fusion proteins (β-penetrenes).

## Materials and Methods

### Sequences

For sequence and structural comparisons of Bunyavirus M encoded proteins representatives of the five genuses were used, including pleboviruses Sandfly fever virus, Sicilian strain (SAN, accession number: AAA75043) and Rift Valley fever virus (RVF, P03518), orthobunyavirus Bunyamwera virus (BUN, NP047212), hantavirus Hantaan virus, strain 76–118 (HAN, P08668), nairovirus Crimean-Congo hemorrhagic fever virus, strain IbAr (CCHF, NP950235), and tospovirus tomato spotted wilt virus, ordinary strain (TSWV, NP049359). Additional phlebovirus M sequences compared included those of Uukuniemi virus (UUK, NP941979) and Punta Toro virus (PTV, VGVUPT). Bunyavirus M sequences were compared to sequences encoded in Alphavirus subgenomic RNA, including structural proteins of Sindbis virus (SIN, P03316), Semliki Forest virus (SFV, NP463458), Venezuelian equine encephalitis virus, strain TC-83 (VEE, P05674), Western equine encephalitis virus, strain McMillan (WEE, AAF60166), O'nyong-nyong virus, strain GULU (ONN, P22056), Mayaro virus, strain TRVL4675 (MAY, AA033335), Barmah Forest virus strain BH2193 (BFV, AA033347) and Ross River virus, strain NB5092 (RRV, NP740686). Comparisons were also made with proteins encoded by RNA2 of the HZ isolate of Rice stripe virus, a Tenuivirus (pvc2, AA031607), and with certain retroviruses of *Caenorhabditis elegans*, including Cer13 (hypothetical protein Y75D11A.5, NP508324). We also compared Bunyavirus M sequences to structural proteins of Flaviviruses, including members of the flavivirus genus tick-borne encephalitis virus, strain Neudoerfl (TBEV, P14336); Japanese encephalitis virus, strain JaOARS982 (JEV, P32886), yellow fever virus, strain 17D-204 (YFV, P19901), dengue virus type 2, strain PR-159/S1 (DEB, P12823), and West Nile virus, strain NY 2000-crow3356 (WNV, AF404756). The prototype hepaciviruses, strain H (subtype 1a) of hepatitis C virus (HCV, P27958), and several pestiviruses, including the Alfort 187 strain of classical swine fever virus, aka hog cholera virus (CSFV, CAA61161), bovine viral diarrhea virus genotype 1 aka pestivirus type 1, stain NADL (BVDV, CAB91847) and border disease virus, strain BD31 (BVD, AAB37578), were used in other comparisons.

### Proteomics computational methods

Methods to derive general models of surface glycoproteins have been described previously [2]. PRSS3, a program derived from rdf2 [19], which uses the Smith-Waterman sequence alignment algorithm [20], was used to determine the significance of protein alignments. PRSS3 is part of the FASTA package of sequence analysis programs available by anonymous ftp from ftp.virginia.edu. Default settings for PRSS3 were used, including the blosum50 scoring matrix, gap opening penalty of 12, and gap extension penalty of 2. MacMolly (Soft Gene GmbH, Berlin) was used to locate areas of limited sequence similarity and to perform Chou-Fasman and Robson-Garnier analyses [21,22]. PHDsec (Columbia University Bioinformatics Center, ) was the preferred method of secondary structure prediction [23]. PHDsec predicts secondary structure from multiple sequence alignments by a system of neural networks, and is rated at an expected average accuracy of 72% for three states, helix, strand and loop. Domains with significant propensity to form transmembrane helices were identified with TMpred (ExPASy, Swiss Institute of Bioinformatics, ). TMpred is based on a statistical analysis of TMbase, a database of naturally occurring transmembrane glycoproteins [24]. Sequences with a propensity to partition into the lipid bilayer were identified with Membrane Protein e**X**plorer version 2.2a from the Stephen White laboratory using default settings [25]. The NetOglyc server  was used to predict mucin type GalNAc O-glycosylation sites. RasMac (University of California Regents/Modular CHEM Consortium, ), developed by Roger Sayle, was used to render a 3D model of SFV E1, which was extrapolated to SIN E1 and SAN GC.

## Results

Similar sequences or common structural/functional motifs are located collinearly in the carboxyl terminal glycoprotein of Sandfly fever virus and Sindbis virus envelope glycoprotein E1.

Previously, Gallaher and coworkers modeled the structure of the retroviral transmembrane glycoprotein (TM) [2] onto the scaffold of the known structure of the HA2 portion of the influenza virus hemagglutinin [26]. Later, Gallaher [1] fit the fusion protein of Ebola virus, a filovirus, to retroviral TM. Both models proved remarkably similar to the structures of these fusion proteins solved later by X-ray crystallography [27-29]. These results indicate that Gallaher's "Rosetta Stone" strategy, which employs the fusion peptide and other identifiable features in combination with computer algorithms that predict secondary structure, is a useful approach to the construction of working models of class I viral fusion proteins. This approach, supplemented with newer proteomics computational tools, was applied to envelope glycoproteins encoded by members of the Bunyaviridae. Our initial finding, obtained using the PRSS3 alignment algorithm [20,30], was that the amino acid (aa) sequence of Gc of Sandfly fever virus (SAN), a phlebovirus, has a significant similarity (p < 0.002) with the aa sequence of E1, the fusion protein of Sindbis virus (SIN), an Alphavirus (Table 1). SAN Gc also showed significant overall alignments with E1 of several other Alphaviruses examined, including Semliki Forest virus (SFV), Western equine encephalitis virus (WEE) and O'nyong-nyong virus (ONN). The Gc proteins of the three other phleboviruses, Rift Valley fever virus (RVF), Uukuniemi virus (UUK) and Punta Toro virus (PTV), the only phleboviruses with completely sequenced Gc coding regions also showed significant sequence similarities to certain Alphavirus E1 proteins. The alignment of RVF Gc with SIN E1 and WEE E1 was statistically significant, but the alignment with SFV E1 was not. PTV Gc showed the highest overall aa sequence similarity with any Alphavirus E1 examined, that of Venezuelan equine encephalitis virus (VEE) (p < 0.0004). UUK Gc showed a significant overall alignment only with Ross River virus (RRV) E1, while RRV E1 failed to align with any of the other three phlebovirus Gc examined. These results from multiple comparisons of phleboviruses Gc and Alphavirus E1 indicate that the significant alignment between SAN Gc and SIN E1 is not a statistical aberration, but may underlie structural and functional similarities between the two viral glycoproteins. It is also of interest that the PRSS3 sequence alignment tool permitted detection of similarities not detected than by the use of BLASTp or related computational methods.

**Table 1:** Comparison of phlebovirus Gc with Alphavirus E1 using the PRSS3 sequence algorithm.

	Alphavirus E1^1^							
Phlebovirus Gc	SIN	SFV	WEE	VEE	MAY	RRV	BFV	ONN
SAN	0.002	0.02	0.001	0.004	0.05	NS	0.03	0.007
RVF	0.04	NS	0.03	0.003	NS	NS	0.04	NS
PTV	NS	0.04	0.0002	0.0004	NS	NS	NS^2^	NS
UUK	NS	NS	NS	NS^3^	NS	0.05	NS	NS

^1^Two-way comparisons were done between the full-length amino acid sequences of the indicated glycoproteins. Probabilities (p values) of a significant alignment are based on 1000 shuffles. SAN: Sandfly fever virus, RVF: Rift valley fever virus; PTV: Punta Toro virus; UUK: Uukuniemi virus; SIN: Sindbis virus; SFV: Semliki Forest virus; WEE: Western equine encephalitis virus; VEE: Venezuelan equine encephalitis virus; MAY: Mayaro virus; RRV: Ross River virus; BFV: Barmah Forest virus; ONN: O'nyong-nyong virus.

^2^p = 0.08

^3^p = 0.07

**Figure 1:**
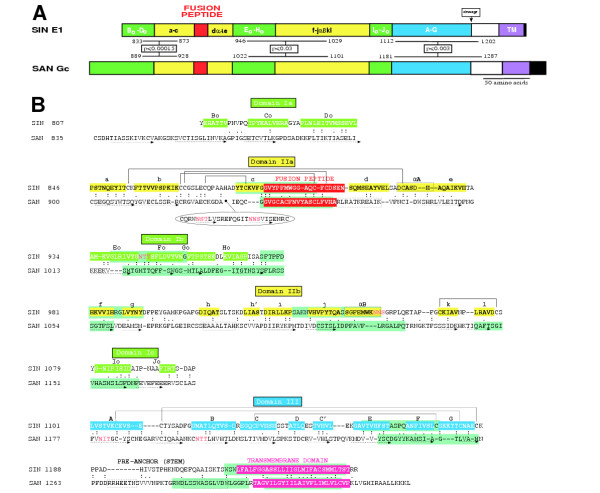
Colinear arrangement of similarities in Sindbis virus E1 and Sandfly fever virus Gc. Alignments were constructed as detailed in the text. Panel A: Linear arrangement of the domain structure of SIN E1 and proposed domain structure of SAN Gc according to the convention for class II viral fusion proteins (β-penetrenes) originally described for TBEV E by Rey et al. [5]. Regions of significant sequence similarities in SIN E1 and SAN Gc determined by the PRSS3 sequence alignment program are indicated. Probabilities (p values) are based on 1000 shuffles. Panel B: Amino acids are numbered from the beginning of the Sindbis virus subgenonic mRNA encoded polyprotein and the beginning of the SAN M segment encoded polyprotein. (:) refers to identical amino acids. (.) refers to chemically similar amino acids. Plum amino acids: N-glycosylation sites. Hydrophobic transmembrane domains were predicted using TMpred. Sequences with significant WWIHS scores were identified by MPeX (olive). In SAN Gc, predicted α-helices are indicated by dashed boxes and predicted β-sheets are underlined with a dashed arrow.

Prior X-ray crystallographic studies have demonstrated that SFV E1 is a class II viral fusion protein (β-penetrene) [6]. Because of its extensive sequence similarity with SFV E1, SIN E1 is assumed to be a class II viral fusion protein [31]. The sequence similarities between SAN Gc and SIN E1 do not permit alignment by computational methods alone. The significance of the overall sequence similarity can, however, be attributed to three collinear similarity regions between the two glycoproteins detected by the PRSS3 algorithm (Fig. 1A). Beginning from the amino terminus, the first sequence similarity starts in β-sheet Do in domain Ia of SIN E1 and extends past β-sheet b in domain IIa. The alignment was significant (P < 0.0002) between aa 889–928 of SAN Gc and aa 833–873 of SIN E1 (Fig. 1A). The next significant sequence similarity, aa 1022–1101 of SAN Gc and aa 946–1029 of SIN E1 (p < 0.03), includes the SIN E1 sequence from β sheet Fo in domain Ib to β sheet i in domain IIb. The third region of similarity, SAN Gc aa 1181–1287 and SIN E1 aa 1112–1202 (p < 0.003), includes most of SIN E1 domain III. In addition, two domains that are characteristic of class II viral fusion proteins are also apparent in SAN Gc. Prior studies have shown that aa 887–904 of SFV E1 are critically involved in virion:cell fusion, and indicate that this region includes the fusion peptide [32,33]. The fusion peptide of SIN E1 is assumed to be located similarly [31]. The fusion peptides of the class II viral fusion proteins are located at the end of domain II, and consist predominantly of aromatic aa (usually phenylalanine [F] or tryptophan [W]), hydrophobic aa, and aa with high turn potential (glycine [G] and proline [P]). Cysteine linkages usually stabilize the fusion peptides of class II viral fusion proteins in the overall structure (Fig 1A, red). A sequence (aa 960–977) corresponding to a consensus class II fusion peptide is present in SAN Gc in a similar location to the SIN E1 fusion peptide (Fig. 1A). Another common domain of class II viral fusion proteins readily identifiable in SAN Gc is the carboxyl terminal transmembrane anchor. Rossmann and coworkers provided experimental evidence that SIN E1 aa 1215–1241 contains the transmembrane domain of SIN E1 [31]. A similar hydrophobic sequence is located near the carboxyl terminus of SAN Gc. TMpred, an algorithm that identifies possible transmembrane helices, assigns a significant score of 3048 (>500 is statistically significant) to aa 1303–1322 of SAN Gc, which suggests that this is the transmembrane anchor of SAN Gc. Using the regions of local similarity and the fusion peptide and transmembrane domains, which are collinear, a proposed alignment between SAN Gc and SIN E1 can be constructed (Fig. 1B). The alignment necessitates only one "insertion." Relative to domain IIb of SIN E1, it appears that SAN Gc, has an added sequence (aa 932–958), a proposed "loop" flanked by cysteines and containing two N-linked glycosylation sites (NXT/S) reminiscent of glycosylated loops of other viral envelope proteins [34].

### Membrane interfacial domains in Bunyavirus glycoproteins

To provide support for the proposed alignment of SAN Gc and SIN E1, another proteomics computational tool was used to compare potential membrane interactive domains in the glycoproteins. Besides fusion peptides, a motif that can be important in virus:cell fusion and is present in many class I and class II viral fusion proteins is an aromatic aa rich domain proximal to the transmembrane anchor [35]. The pre-anchor domains are not highly hydrophobic according to the Kyte-Doolittle hydropathy prediction algorithm, but have a tendency to disrupt and partition into bilayer membranes as revealed by analyses using the Wimley-White interfacial hydrophobicity scale (WWIHS) [35,36]. SIN E1 contains a sequence prior to and overlapping the transmembrane anchor with a significant WWIHS score as determined by Membrane Protein eXplorer (MPeX) [25]. SAN Gc has two sequences with significant WWIHS scores in this region, the pre-anchor and the putative transmembrane domain. The fusion peptides of all class I and II viral fusion proteins examined to date overlap sequences with significant WWIHS scores (RFG, unpublished observation). The proposed fusion peptide in SAN Gc consists of a sequence with a significant WWIHS score, which further supports the assignment of this sequence as the fusion peptide. Additional sequences with significant WWIHS scores are located collinearly along SAN Gc and SIN E1. In total, six of the seven sequences in SAN GC with significant WWIHS scores overlap in the proposed alignment with sequences with significant WWIHS in SIN E1. Analysis of membrane interfacial potential in the primary sequences thus provides further support for the proposed alignment of SAN Gc and SIN E1.

### A model of phlebovirus Gc

**Figure 2:**
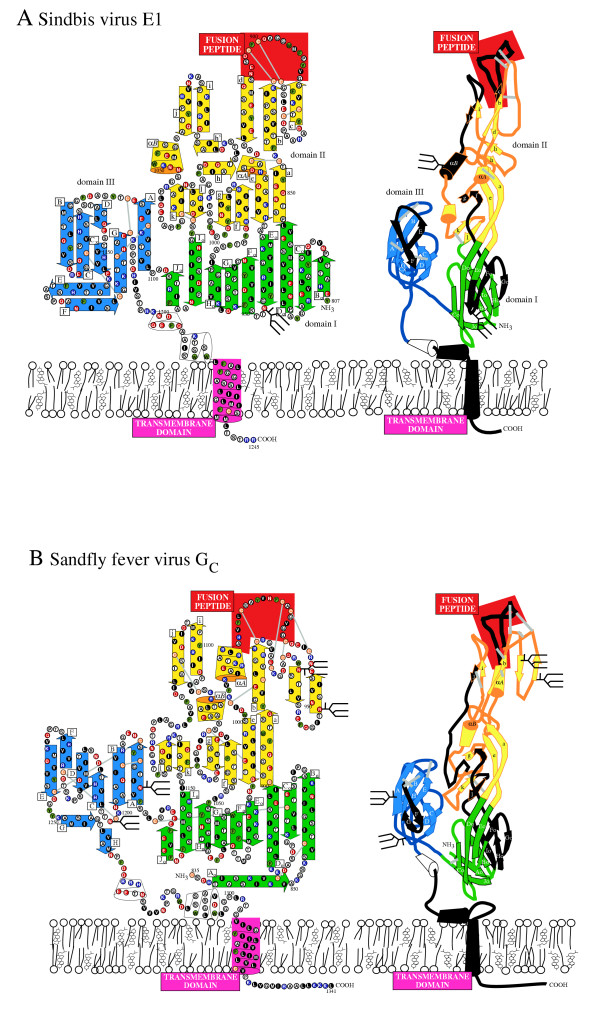
Model of Sandfly fever virus Gc based on predicted structure of a Sindbis virus E1 fusion intermediate. Panel A: A structural intermediate of SFV E1 as determined by Gibbons et al. [33] was projected to SIN E1. Panel B: A model fitting SAN GC to the predicted structure of SIN E1. Structures predicted to be similar are color-coded the same way in SIN E1 and SAN Gc. Grey lines: dicysteine linkages. Black stick figures: N-glycosylation sites (sites with central proline are often not used). Regions with significant Wimley-White interfacial hdrophobicity scale scores were predicted with MpeX (black).

Recently, Gibbons and coworkers determined the structure of a fragment of the SFV E1 ectodomain (lacking carboxyl terminal aa 392–438) after exposure to low pH and liposomes [33]. Under these conditions, which mimic an endosomal environment, the SFV E1 ectodomain fragment changes from a soluble monomer to a trimer as it inserts into the liposomal membrane after exposure to low pH. A similar post-fusion structure was found in two other class II fusion proteins, E of DEN and TBEV [10,11]. These investigators proposed several possible fusion intermediates of SFV E1 and other class II viral fusion proteins after exposure to low pH. These intermediates are assumed to be similar to structural intermediates of SIN E1. To determine the plausibility of the proposed SAN Gc and SIN E1 alignment, a model of SAN Gc was scaffolded on a presumed structural intermediate of SIN E1 in which compared to the orientation in the virion at neutral pH, domain III is displaced closer to the fusion peptide (Fig. 2). The collinear sequence alignments between SAN Gc and SIN E1 suggest that both glycoproteins may have a similar domain structure. Similar sequences/structures are drawn in similar locations. In this possible fusion intermediate, the putative SAN Gc fusion peptide is assumed to be located at the end of the molecule furthest from the carboxyl terminal (C-terminal) transmembrane anchor domain. Like SIN E1 and other class II fusion proteins SAN Gc may be comprised mostly of antiparallel β sheets, an expectation supported by several secondary structure prediction algorithms, including PHDsec [23], Chou-Fasman [21] and Robson-Garnier [22] analyses. The proposed SAN Gc structure conforms closely to the structural predictions of PHDsec, the most robust algorithm, but is also generally consistent with both Chou-Fasman and Robson-Garnier predictions. In some cases because of significant aa sequence similarity with SIN E1, ambiguous structures in SAN Gc are depicted as in SIN E1. Additional evidence for the proposed alignment is the location of cysteine residues of SAN Gc and SIN E1. The cysteine residues are usually the most conserved aa in within a protein family because disulfide linkages are a critical determinant of secondary structure. The dicysteines of SIN E1 (Fig. 2B) are arranged such that bonds are formed only between residues within the same putative domain. A similar arrangement is feasible for SAN Gc with C residues present in close proximity after scaffolding on the SIN E1 structure, and with possible linkages occurring within the three proposed domains. This model also locates the four SAN Gc glycosylation sites so they are surface accessible.

There are many possible alternatives to the cysteine linkages and secondary structures of SAN Gc drawn in Figure 2. Nevertheless, a plausible three-dimensional model of SAN Gc that conforms to the scaffold of the known structure of Alphavirus E1 can be constructed. This result coupled with predictions of a predominantly β sheet secondary structure of SAN Gc provides further support its proposed alignment with SIN E1.

### Sequence/structural features of Bunyavirus Gc suggest that a class II fusion protein structure is conserved in members of the Bunyaviridae

To provide additional evidence for the proposed SIN E1/SAN Gc alignment and the SAN Gc class II fusion protein model, we determined whether structural/sequential similarities with class II fusion proteins are conserved in envelope proteins encoded by other members of the Bunyavirus family. With the exception of tospoviruses, that use an ambisense strategy for synthesis of a nonstructural protein, Bunyavirus M segments are negative in polarity and the mRNA transcribed contains a long open reading frame [37]. The M segment mRNA is translated as a polyprotein, which is post-translationally processed [38]. There is considerable diversity in the number and sizes of the M segment encoded polyproteins produced in infected cells, but all Bunyaviruses encode at least two glycoproteins, Gn and Gc. Prior analyses have revealed similarities between Gc encoded by members of orthobunyaviruses and tospoviruses [18,39], but evidence that Gn and Gc serve analogous functions in each Bunyavirus genus has not been available previously. Comparisons among Gc of members of the five genuses of the Bunyaviridae using the PRSS3 algorithm revealed that the type members of each genuses display significant sequence similarities with certain Gc of viruses of other Bunyavirus genuses (Table 2). The most significant alignment detected among members of different genuses of the Bunyaviridae, was between RVF, type member of phleboviruses, and tomato spotted wilt virus (TSWV), type member of tospoviruses (p < 10^-18^). As noted previously [18,39], orthobunyavirus Gc also show a significant similarity to tospovirus Gc, with Bunyamwera virus (BUN) Gc displaying a significant similarity to TSWV Gc (p < 10^-8^). As with phlebovirus Gc, the prototype of the hantavirus genus, Hantaan virus (HAN), showed a modest sequence alignment (p < 0.05) with SIN E1, further supporting the proposed similarities between Bunyavirus Gc and Alphavirus E1. Significant alignments were not detected between Bunyavirus Gc or Alphavirus E1 and TBEV E or other flavivirus class II viral fusion proteins. Limited local similarities were observed between some Bunyavirus Gc and pestivirus E2. It is noteworthy that the significance of the overall sequence similarities between certain phlebovirus Gc and Alphavirus E1 is higher than some similarities among Gc of some prototypic members of the Bunyaviridae (compare Table 1 and 2). Collectively, these results suggest that Gc of Bunyaviruses share a limited number of similar sequences.

**Table 2:** Similarities among Bunyavirus Gc, Alphavirus E1 and related glycoproteins as determined with the PRSS3 sequence algorithm.

	Viral protein^1^							
Viral protein	Hanta HAN Gc	Nairo CCHF Gc	Phlebo RVF Gc	Tospo TSWV Gc	Alpha SIN E1	CeRV Cer13 Env^2^	Tenui RiSV pvc2^2^	Flavi TBEV E
Obuny BUN Gc	0.0005	0.01	0.009	10^-5^	NS	NS	NS	NS
Hanta HAN Gc	---	0.0001	NS	NS	0.05	NS	0.003	NS
Nairo CCHF Gc	---	---	0.05	0.0001	NS	NS	NS	NS
Phlebo RVF Gc	---	---	---	10^-18^	0.04	10^-8^	0.001	NS
Tospo TSWV Gc	---	---	---	---	NS	NS	NS	NS
Alpha SIN E1	---	---	---	---	---	0.02	NS	NS
CeRV Cer13 Env^2^	---	---	---	---	---	---	0.02	NS
Tenui RiSV pvc2^2^	---	---	---	---	---	---	---	NS

Two-way comparisons were done between the full-length amino acid sequences of the indicated glycoproteins. Probabilities (p values) of a significant alignment are based on 1000 shuffles. BUN: Bunyamwera virus; HAN: Hantavirus; CCFV: Crimean-Congo hemorrhagic fever virus; RVF: Rift valley fever virus; TSWV: Tomato spotted wilt virus; SIN: Sindbis virus; Cer13: *Caenorhabditis elegans *retrovirus 13; RiSV; rice stripe virus; TBEV: tick-borne encephalitis virus.

^2^C-terminal sequence.

**Figure 3:**
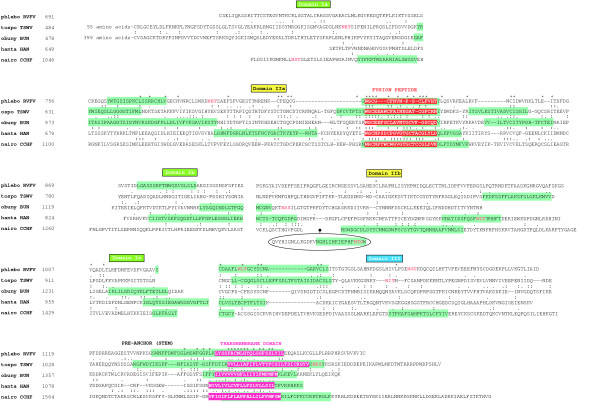
Alignment of Gc amino acid sequences of prototype members of the five genuses of the Bunyaviridae family. Alignments were constructed by identifying the fusion peptide (red) and the transmembrane anchor (violet) as described in the text. Additional local sequence similarities were identified by using the Complign feature of MacMolly, the PRSS3 alignment algorithm or by inspection. Sequences with significant WWIHS scores (olive) were identified by MPeX.

Available computational methods alone do not permit overall alignments of Bunyavirus Gc, however, through the use of PRSS3 and MacMolly alignment tools and by inspection certain common sequences can be identified. The most highly conserved sequences among type members of the five Bunyavirus genuses conform to a consensus class II fusion peptide (Fig. 3, red) and to a carboxyl terminal transmembrane domain (Fig. 3, violet). Certain cysteine clusters, which are likely to stabilize the secondary structures of the proteins, also are present in similar locations along the proteins, including the beginning of putative domain III (Fig. 3). Further support for the alignment of Bunyavirus Gc is obtained by the use of the WWIHS. Sequences with significant WWIHS scores are located in similar locations in Bunyavirus Gc (Fig. 3, olive). The proposed fusion peptides and transmembrane domains of each of the Bunyavirus Gc examined displayed significant WWIHS scores. In addition, most of the Gc examined had sequences with significant WWIHS scores in the putative IIa domains and either the putative Ic domain or the beginning of adjacent domain III. This alignment suggests that orthobunyaviruses and tospoviruses Gc have extended regions of approximately 400 and 50 amino acids at the amino terminus relative to phlebovirus, hantavirus and nairovirus Gc. These results also suggest that motifs involved in virion:cell fusion are conserved in Gc throughout the Bunyaviridae, and that Gc is the fusion protein encoded by members of each Bunyavirus genus.

### Sequence/structural features of Bunyavirus Gc are present in a Tenuivirus protein and in an Env encoded by a *Caenorhabditis elegans *retrovirus

**Figure 4:**
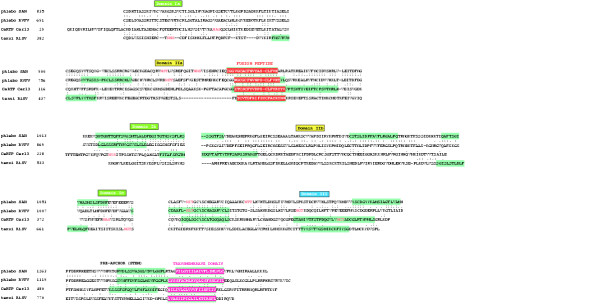
Alignment of phlebovirus Gc amino acid sequences with Tenuivirus surface protein pvc2 and the carboxyl terminal Env protein of a *Caenorhabditis elegans *retrovirus. Sequences are color-coded as in Figure 3.

As previously reported, phlebovirus Gc have a significant similarity to surface protein pvc2 encoded by plant viruses of the Tenuiviridae [40]. The PRSS3 sequence alignment algorithm confirms this similarity (Table 2, p < 0.001). An envelope protein (Env) encoded by a group of retroviruses of the nematode *C. elegans *also demonstrate significant similarities to phlebovirus Gc [41,42]. There is a significant alignment detected by the PRSS3 algorithm between SAN Gc and the carboxyl terminal region of Env of Cer13, a potentially replication-competent member of this group (Table 2, p < 10^-8^). These results further validate the use of the PRSS3 algorithm to identify limited similarities amongst viral proteins. Alignment of the carboxyl terminal portion of the pvc2 protein of rice stripe virus (RiSV), a Tenuivirus, and the envelope protein encoded by Cer13 retrovirus with two phlebovirus Gc reveals a collinear arrangement of fusion peptide consensus sequences (Fig. 4, red) and potential carboxyl terminal transmembrane domains (Fig. 4, violet). These proteins also have several overlapping sequences with significant WWIHS scores (Fig. 4, olive). The retention of these features in proteins encoded by evolutionarily distant genomes, provides further evidence that these motifs are important for the function of Bunyavirus fusion proteins.

### Protein order in polyproteins encoded by Bunyaviridae M segments

**Figure 5:**
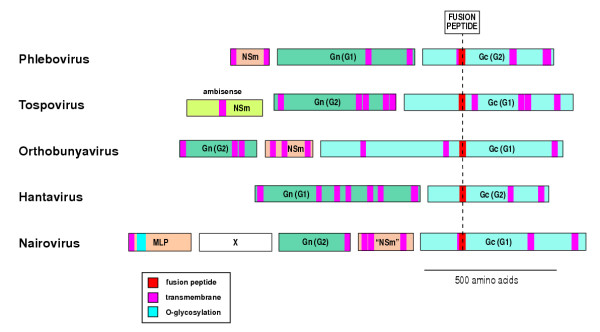
Common order of proteins in Bunyavirus M segment polyproteins. Related glycoproteins Gn and Gc are in the same order in the polyproteins of prototypic members of the Bunyaviridae. Prior designations of the glycoproteins are indicated in parentheses. Hydrophobic domains were predicted using TMpred. The O-glycosylation rich (mucin-like) region in CCHF was delineated using NetOGlyc 3.1 as described previously by Sanchez and coworkers [46]. These authors also described the indicated potential cleavages of the CCHF polyprotein.

The longest open reading frames of M segments of all members of the Bunyaviridae are antisense to the virion RNA. mRNAs transcribed from Bunyavirus M segments are translated into large polyproteins that are subsequently cleaved by into functional proteins [38,43]. Gc of members of each of the five Bunyavirus genuses are the carboxyl terminal proteins of the polyprotein (Fig. 5). Because viral proteins with similar functions may have similar genome locations, we sought evidence for sequence similarities among other Bunyavirus proteins encoded by the M segment. Gn of type members of the five Bunyavirus genuses were compared to each other and each had a limited sequence similarity to at least one other Gn of a type member of a different genus (Table 3). The most significant alignment was between the Gn proteins of HAN, a hantavirus, and CCHF, a nairovirus (p < 10^-4^). HAN Gn also showed a significant alignment with Gn of RVF, a phlebovirus. Both HAN Gn and Gn of TSWV, a tospovirus, also showed significant alignments with envelope protein 2 (E2) of SIN. SIN E2 has been implicated as the virion protein responsible for binding to the cell surface receptor [44]. These results suggest that the Gn of Bunyaviruses have limited similarity, and may have a common role or roles in the virus replication cycle. The order (from amino to carboxyl terminus) of proteins in the polyproteins of SIN and other members of the Alphaviridae is Capsid-E2-E3-6K-E1. Receptor and fusion functions may reside in two different Bunyavirus proteins, Gn and Gc respectively, occurring in the same order as the envelope glycoproteins, E2 and E1, that carry out these functions in Alphaviruses (Fig. 5). The similarities in protein order and functions support the hypothesis that Gc is the fusion protein of Bunyaviruses. These results also support the suggested nomenclature of Gn and Gc for the Bunyavirus M segment encoded glycoproteins as a replacement for the current ambiguous nomenclature, which variously assigns the designation G1 or G2 to unrelated Bunyavirus glycoproteins. The presumptive protein encoded by the amino terminal region of the pvc2 protein of RiSV also showed limited similarity to SIN E2 (Table 3). Thus, the similarities between proteins encoded by a Tenuivirus extend to another glycoprotein of a virus with a class II fusion protein.

**Table 3:** Similarities among Bunyavirus Gn, Alphavirus E2 and related glycoproteins as determined with the PRSS3 sequence algorithm.

	Viral protein^1^							
Viral protein	Hanta HAN Gn	Nairo CCHF Gn	Phlebo RVF Gn	Tospo TSWV Gn	Alpha SIN E2	CeRV Cer13 Env^2^	Tenui RiSV pvc2^2^	Flavi TBEV E
Obuny BUN Gn	NS	NS	NS	0.02	NS	NS	NS	NS
Hanta HAN Gn	---	10^-4^	0.03	NS	0.05	NS	NS	NS
Nairo CCHF Gn	---	---	NS	NS	NS	NS	NS	NS
Phlebo RVF Gn	---	---	---	NS	NS	NS	NS^3^	NS
Tospo TSWV Gn	---	---	---	---	0.04	NS	NS	NS
Alpha SIN E2	---	---	---	---	---	NS	0.04	NS
CeRV Cer13 Env^2^	---	---	---	---	---	---	0.025	NS
Tenui RiSV pvc2^2^	---	---	---	---	---	---	---	NS

^1^Two-way comparisons were done between the full-length amino acid sequences of the indicated glycoproteins. Probabilities (p values) of a significant alignment are based on 1000 shuffles. BUN: Bunyamwera virus; HAN: Hantavirus; CCFV: Crimean-Congo hemorrhagic fever virus; RVF: Rift valley fever virus; TSWV: Tomato spotted wilt virus; SIN: Sindbis virus; Cer13: *Caenorhabditis elegans *retrovirus 13; RiSV; rice stripe virus; TBEV: tick-borne encephalitis virus.

^2^N-terminal sequence.

^3^p = 0.08

The simplest M polyprotein, encoding only Gn and Gc, is that of hantaviruses (Fig. 5). In addition to Gn and Gc, phlebovirus and orthobunyavirus encode nonstructural proteins (NSm) that have two or three potential transmembrane spanning domains as detected by TMpred (Fig. 5). Nairoviruses, such a Crimean-Congo hemorrhagic fever virus (CCHF), synthesize a similar protein [45,46], and we have designated this protein "NSm." NSm of the RVF and BUN, type members of phlebovirus and orthobunyaviruses, as well as the "NSm" protein of CCHF have short regions of similarity with each other as revealed by the PRSS3 sequence alignment algorithm, although the overall alignments are not significant. There were also short regions of similarity between the NSm proteins of these three Bunyavirus genuses and the prM/M-like proteins of Flaviviruses (not shown). Immature virions of members of the flavivirus genus of the Flaviviridae contain a precursor prM to the small membrane protein M. prM is cleaved by furin or by a furin-like protease during virus release to produce the mature M protein localized on the surface of flavivirus virion [47,48]. Flavivirus PrM/M, contains two potential membrane spanning domains, and their functions may include shielding of internal cellular membranes from the fusion peptide of E [7,47]. It is possible that the phlebovirus, orthobunyavirus and nairovirus M segment encoded nonstructural proteins, all with multimembrane-spanning potential, serve the same function for Gc. NSm of TSWV, a tospovirus, showed no sequence similarity or structural similarity with any Bunyavirus protein examined. The functions of tospovirus NSm, which is encoded by the only positive polarity gene in any M segment, and the other Bunyavirus NSm proteins remain to be determined. Nairovirus M may encode two additional proteins, a mucin-like protein (MLP), which contains a variable region with a high concentration of potential O-glycosylation sites, and a protein designed here X, neither of which have obvious homologs encoded by members of the other Bunyavirus genuses [45,46]. The coding sequences of Bunyavirus M appear to have evolved in a manner preserving the order of the glycoproteins Gn and Gc, while allowing for insertion or deletion of sequences encoding additional proteins.

## Discussion

Proteomics computational analyses suggest that Bunyavirus Gc proteins are class II viral fusion proteins (β-penetrenes), with a structure similar to the fusion proteins of Alphaviruses and Flaviviruses. Similar sequences or common structural/functional motifs are collinearly located in Bunyavirus Gc and Alphavirus E1. Features common to other class II fusion proteins, including an internal fusion peptide, a carboxyl terminal transmembrane domain and regions with a high propensity to interface with bilayer membranes, are conserved and in similar locations in Gc of viruses in each genus of the Bunyaviridae. These features are also present in glycoproteins encoded by nonenveloped Tenuiviruses of plants, and a group of *C. elegans *retroviruses previously shown to have remarkable sequence similarities to phlebovirus Gc. These results also indicate that Gallaher's "Rosetta Stone" strategy can be used to identify potential class II viral fusion proteins, as demonstrated previously for class I fusion proteins [1-3,49]. The common placement of proper names or "cartouches" allowed the ancient languages of the Rosetta Stone to be deciphered. As advanced by Gallaher, fusion peptides can serve a similar function to facilitate alignment of viral fusion proteins with limited sequence similarities.

Many viral fusion proteins fit neither class I or II and it is likely that other classes of viral fusion protein also exist. However, among major classes of enveloped RNA viruses, there are at least six, myxoviruses, retroviruses, paramyxoviruses, filoviruses, arenaviruses and coronaviruses, that encode class I viral fusion proteins [1-4]. Alphaviruses, members of the flavivirus genus of the Flaviviruses, and according to current analyses, Bunyaviruses, encode class II viral fusion proteins. Computational analyses suggest that members of the two other Flavivirus genuses, hepaciviruses and pestiviruses, encode variant class II fusion proteins [12]. The viruses encoding class II or II viral fusion proteins thus represent a substantial portion of enveloped RNA virus families known to infect vertebrates. It is significant that representative class I and II encoding viruses are also found in evolutionarily distinct plant viruses and viruses or virus-like genomic elements of nematodes and insects [41,42,50,51]. There may be constraints on the structures of viral proteins capable of effectively mediating virion:cell fusion, or a limited number of enveloped RNA virus lineages.

Alphaviruses appear to use separate envelope proteins for fusion (E1) and attachment (E2) [44]. Because Bunyavirus Gc display similarities to Alphavirus E1 and certain Bunyavirus Gn display limited sequence similarities to Alphavirus E2, Bunyaviruses may have adapted a similar strategy. Verification that Gc is the fusion protein of Bunyaviruses will require a combination of X-ray crystallographic structural studies and site-directed mutagenesis of key features such as the putative fusion peptide. Verification that Gn serves as the receptor binding protein for any Bunyavirus requires identification of its cell surface receptor. E, the class II fusion protein of TBEV, dengue virus, and other members of the flavivirus genus of the Flaviviridae, mediates both virion:cell fusion and receptor-binding [52,53]. Therefore, it is possible that Bunyavirus Gc serves both as the fusion protein and receptor binding protein.

The remarkable similarities in both the pre- and post-fusion forms of the fusion proteins of SFV E1, an Alphavirus, and DEN and TBEV, members of the flavivirus genus of the Flaviviruses, in the absence of detectable sequence similarities, suggest that Alphavirus and Flavivirus class II fusion proteins may have diverged from a common progenitor. Alternatively, there may have been convergent evolution towards the common structure. Likewise, the sequence similarities detected between phlebovirus Gc and SIN E1 are consistent with divergent evolution from a common progenitor, but are insufficient to directly establish a phylogenic relationship. The results presented here suggest that Gc of members of the Bunyaviridae may have a common ancestor. Gn and Gc are in analogous locations in the polyproteins encoded by the five genuses of the Bunyaviridae. The simplest Bunyavirus M polyprotein, that of hantavirus members, encodes only Gn and Gc, whereas M of members of other Bunyavirus genuses encode several additional proteins. Therefore, divergence of Bunyavirus M segments may have occurred either through acquisition of sequences and/or lose of sequences in a cassette manner constrained in part by the locations of the major glycoproteins.

**Figure 6:**
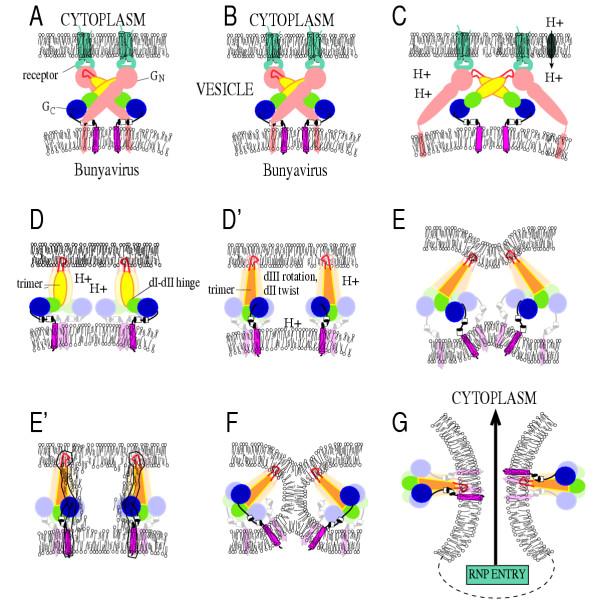
Hypothetical model of Bunyavirus:cell fusion. Steps in the entry process of Bunyaviruses can be extrapolated from current models of class II viral fusion protein-mediated virion cell fusion. Panel A. The Bunyavirus glycoproteins Gn and Gc are modeled according to SIN virion structure analyses by Zhang et al. [31]. Based on limited similarities with Alphavirus E2 proteins (Table 3), Gn is depicted as the receptor-binding protein of Bunyaviruses. Certain Bunyaviruses may encode other membrane-associated proteins that interact with the fusion peptide or other regions of Gc. Panel B: Receptor-binding triggers uptake of Bunyavirus virion by endocytosis. Panel C: Acidification of the endocytic vesicle occurs via the action of proton transporters and may initiate Gn and Gc dissociation. Panel D: bending at the flexible "hinge" region beween domains I and II permits Gc trimer formation and insertion of the fusion peptide into the endosomal vesicle membrane. Panel D' Alternatively, Gc trimer formation may involve the rotation of domain III and a rearrangement (twist) of domain II as shown for SFV E1, DEN E and TBEV E [11,33,54]. Panel E: As previously proposed [11,33,54] the formation of more extensive Gc contacts in the trimers and stem regions may release of energy for distortion of the endosomal and viral membranes resulting in formation of "nipple-like" projections. Panel E': Alternatively, aa sequences of Gc that form a track with the ability to interface with bilayer membranes (Fig. 2, black), may facilitate mixing of the endosomal and viral membranes. Panel F: Formation of further trimer contacts and hemifusion. Hemifusion may not occur in the D' and E' pathway. Panel G: Formation of the "fusion pore" and entry of the ribonucleoprotein (RNP) segments. Modified from models and concepts proposed in references 9-12.

Comparisons of divergent viral fusion proteins with internal fusion peptides can reveal features essential for virion:cell fusion. Regions of high membrane interfacial propensity including the fusion peptide and the transmembrane anchor, appear in similar locations in Bunyaviruses, Alphaviruses and Flaviviruses. The presence of several additional sequences with the propensity to interact with bilayer membranes in class II viral fusion proteins has not been considered in previous virion:cell fusion models [9,11,54]. Cell entry of Alphaviruses and Flaviviruses is believed to occur via the endocytic route, and it is likely that this is the entry route of Bunyaviruses [55]. Following binding to the cellular receptor, a putative function of Bunyavirus Gn (Fig. 6A), the Bunyavirus virion may be taken up in an endocytic vesicle (Fig. 6B). Exposure to acidic pH in the endosome may trigger conformational changes in the envelope proteins and in the virion itself resulting in dissociation of Gn and Gc (Fig. 6C). Current models of fusion mediated by Alphavirus and Flavivirus class II viral fusion proteins suggest that the low pH of the endosome triggers trimerization and a bending of class II fusion proteins at a flexible "hinge" region between domains I and II elevating the fusion peptide so that it can insert into the host membrane [6,9,11,54]. Current models then suggest that a rearrangement of the stem (pre-anchor region), so that there are more extensive interactions with domains I-III, results in a deformation of the viral and target membranes and the formation of apposing membrane "nipples" (Fig. 6E). Subsequently, the nipples are brought closer together by continued interactions of the stem with domains I-III, which results in bilayer hemifusion (Fig. 6F). Complete fusion follows allowing entry of the ribonucleoproteins containing the viral genomic RNA (Fig. 6G). An analogous mechanism, involving deformation and nipple formation of the viral and cellular membranes caused by rearrangements of the viral fusion proteins (six helix bundle formation) has been proposed for class I viral fusion proteins [54].

Current fusion models do not consider that the transmembrane domain and fusion peptide, while anchored into the viral and cellular membranes, would still be free to move laterally without distorting the membranes. More importantly, the virion is quite small compared to the cell, and would be freely mobile. Rearrangement of the fusion proteins may simply draw the virus closer to the cell without distorting either the viral or cellular membranes. An alternative to the models involving apposing membrane nipple formation is suggested by the observation that sequences of class II viral fusion proteins, including the fusion peptide, the transmembrane anchor and other sequences with high WWIHS scores, potentially form a nearly continuous track of membrane interactive regions that could channel the movement of lipids during virion:cell fusion (Fig. 2, black). Similar nearly contiguous sequences with significant WWIHS scores are present in the post-fusion intermediates of Alphaviruses SIN and SFV, DEN and other flaviviruses, and the proposed structures of hepaciviruses and pestiviruses [12]. An intermediate, with the track of sequences with high membrane interfacial propensity, may be the first intermediate formed after exposure to the low pH in liposomes (Fig. 6D'). Upon formation of higher multimers of trimers, the regions with high WWIHS scores, in conjunction with the fusion peptide and transmembrane could then form a "pore" in which the lipids of the cellular and viral bilayer membranes could mix directly (Fig. 6E'). With lipid mixing facilitated by these membrane interfacial sequences bilayer fusion may proceed without a hemifusion step, but still permitting entry of the genome-containing RNP (Fig. 6G).

In the absence of structural determinations by X-ray crystallography, models such as proposed here can provide useful hypotheses to guide experimental strategies for development of vaccines or drugs to prevent or treat infection by viruses with class II fusion proteins. Prior to the availability of X-ray structural data, several potent HIV-1 TM inhibitors were developed [56,57] based on the Gallaher HIV-1 TM fusion protein model [2]. Fuzeon™ (DP178; T20 enfuvirtide), one of these peptides corresponding to a portion of an α helices and the pre-anchor domain, has been shown to substantially reduce HIV-1 load in AIDS patients, and has been approved for use in the treatment of HIV infection in the United States and European Union [58,59]. Peptides targeted to membrane interactive motifs block virion:cell fusion mediated by DEN and West Nile virus, flaviviruses with class II fusion proteins (Hrobowski et al., submitted). Peptide inhibition strategies targeted to Gc may be broadly applicable to various members of the Bunyaviridae.

## Competing Interests

The authors declare that they have no competing interests.

## Authors' Contributions

CEG performed the sequence alignments and assisted in the preparation of figures. RFG supervised the work and wrote the manuscript.
